# HIV and syphilis testing behaviors among heterosexual male and female sex workers in Uganda

**DOI:** 10.1186/s12981-020-00306-y

**Published:** 2020-08-01

**Authors:** Richard Muhindo, Andrew Mujugira, Barbara Castelnuovo, Nelson K. Sewankambo, Rosalind Parkes-Ratanshi, Juliet Kiguli, Nazarius Mbona Tumwesigye, Edith Nakku-Joloba

**Affiliations:** 1 Department of Nursing, College of Health Sciences, Makerere University, Kampala, Uganda; 2 Infectious Diseases Institute, Kampala, Uganda; 3 School of Public Health, College of Health Sciences, Makerere University, Kampala, Uganda; 4 School of Medicine, College of Health Sciences, Makerere University, Kampala, Uganda; 5 Cambridge Institute of Public Health, University of Cambridge, Cambridge, UK; 6 Ward 12/STD Clinic, Mulago Hospital, Kampala, Uganda

**Keywords:** HIV, Syphilis, Testing, Male sex workers, Uganda

## Abstract

**Background:**

In Sub-Saharan Africa where HIV disproportionately affects women, heterosexual male sex workers (HMSW) and their female clients are at risk of acquiring or transmitting HIV and other STIs. However, few studies have described HIV and STI risk among HMSW. We aimed to assess and compare recent HIV and syphilis screening practices among HMSW and female sex workers (FSW) in Uganda.

**Methods:**

Between August and December 2019, we conducted a cross-sectional study among 100 HMSW and 240 female sex workers (FSW). Participants were enrolled through snowball sampling, and an interviewer-administered questionnaire used to collect data on HIV and syphilis testing in the prior 12 and 6 months respectively. Integrated change model constructs were used to assess intentions, attitudes, social influences, norms and self-efficacy of 3-monthly Syphilis and 6-monthly HIV testing. Predictors of HIV and syphilis recent testing behaviors were estimated using negative binomial regression.

**Results:**

We enrolled 340 sex workers of whom 100 (29%) were HMSW. The median age was 27 years [interquartile range (IQR) 25–30] for HMSW and 26 years [IQR], (23–29) for FSW. The median duration of sex work was 36 and 30 months for HMSW and FSW, respectively. HMSW were significantly less likely than FSW to have tested for HIV in the prior 12 months (50% vs. 86%; p = 0.001). For MSW, non-testing for HIV was associated with higher education [adjusted prevalence ratio (aPR) 1.66; 95% confidence interval (CI) 1.09–2.50], poor intention to seek HIV testing (aPR 1.64; 95% CI 1.35–2.04), perception that 6-monthly HIV testing was not normative (aPR 1.33; 95% CI 1.09–1.67) and low self-efficacy (aPR 1.41; 95% CI 1.12–1.79). Not testing for syphilis was associated with low intention to seek testing (aPR 3.13; 95% CI 2.13–4.55), low self-efficacy (aPR 2.56; 95% CI 1.35–4.76), negative testing attitudes (aPR 2.33; 95% CI 1.64–3.33), and perception that regular testing was not normative (aPR 1.59; 95% CI 1.14–2.22).

**Conclusions:**

Non-testing for HIV and syphilis was common among HMSW relative to FSW. Future studies should evaluate strategies to increase testing uptake for this neglected sub-population of sex workers.

## Background

There is global recognition that sex work is a key driver of HIV and other sexually transmitted infections (STI) in the general population [1–4]. Sex workers are female, male and transgender adults and young people who receive money or goods in exchange for sexual services [5]. While most of the evidence relates to female sex workers (FSW) [3, 4], male sex workers (MSW) are increasingly being recognized as a key population contributing to the global burden of HIV and STI [5–7]. Little is known about the risk of HIV acquisition among heterosexual male sex workers (HMSW) but overall, the risk in 2018 for sex workers was 21 times higher than adults aged 15–49 years, and 22 times more likely among gay men and other men who have sex with men than all adult men [6]. HIV prevalence estimates among MSW and female sex workers (FSW) are comparable. Research has found a high HIV prevalence among MSW: 5–31% in North America [7–9], 11.4–23% in South America [10, 11], and 14.5–43.6% in India [12–14]. The HIV burden among MSW is high in sub-Saharan Africa (SSA), with an HIV prevalence of 26.3% and 50% in Kenya and Cote d’Ivoire, respectively [15, 16]. This is comparable to HIV prevalence observed among FSW (36.9%) in this setting [3], and globally (11.8–30.7%) [3]. However, most of these studies focus on MSW whose clients are men and FSW. Of the 800,000 new HIV infections that occurred in Eastern and Southern Africa in 2018, 25% were contributed by MSW,FSW and other key populations [17]. Sex workers are a bridge population; up to 15% of HIV infections in the general population are attributed to sex work [4].

Limited data are available on HMSW, perhaps because HMSW are usually less visible than FSW [18] and the assumption that sex work by heterosexual men constitutes a small proportion of male commercial sex [19, 20]. Additionally, risk of HIV acquisition through insertive penile-vaginal intercourse is lower than that for insertive or receptive anal intercourse among HMSW who have sex with women [21]. However, in SSA where HIV disproportionately affects women, heterosexual MSW and their female clients are at risk of either acquiring or transmitting HIV [22]. Inconsistent condom use, anal sex, lack of access to healthcare services and restrictive policies act in synergy to increase risk of HIV and STI among MSW. Further, STI co-infection increases HIV acquisition and transmission risk [23, 24]. STI like syphilis, chlamydia, trichomoniasis and gonorrhoeae are curable if detected and treated [1, 25]. Effective STI control among key populations is associated with a decline in STI incidence in the general population, and syphilis seroprevalence among FSW is an important proxy indicator of progress in STI control [25]. The World Health Organization (WHO) recommends STI screening for sex workers every 3 months and HIV testing every 6–12 months [26, 27]. Uptake of regular STI and HIV screening services is an important entry point for antiretroviral treatment (ART) and prevention services (oral pre-exposure prophylaxis and voluntary medical male circumcision) [2, 26].

The hidden HMSW sub-population in Uganda is not well described. Anecdotal evidence (field reports and newspaper articles) suggests the existence of HMSW [28]. In northern Uganda, a community leader identified HMSW who target wealthy women as a key challenge to HIV epidemic control [28]. However, no published data are available on STI and HIV testing practices among HMSW in Uganda. Despite paucity of data on STI testing practices, up to 86% of FSW in Uganda report taking an HIV test in the prior 12 months [29, 30]. Our study aimed to assess and compare recent HIV and syphilis screening practices among HMSW, relative to FSW, in selected urban centers in Uganda.

## Methods

### Study design and setting

Between August and December 2019, we conducted a cross-sectional survey of 100 HMSW and 240 FSW in Kampala, the capital city of Uganda, and Mbarara municipality in Western Uganda (combined population 1702,274) to describe recent HIV and syphilis screening practices. Study participants included heterosexual men and women ≥ 17 years engaged in sex work according to self-report of selling sex for goods or money for at least 6 months. A two-stage sampling design was used to recruit study participants.

### Population and procedures

Before recruiting respondents, we conducted a mapping exercise to gain an understanding of typologies, hot spots, network connections and territorial management in Kampala City and Mbarara Municipality as previously reported [30]. We observed from the mapping exercise that forms and strategies for male sex work included brokers, online advertising, social media and recreational venues. In contrast, FSW sold sex on the street and in lodges, bars/clubs and brothels (mainly in Kampala). We first identified three HMSW through key informants (FSW, escort service manager and bar/club maids). They were given information about study aims and recruitment of HMSW and provided with two to three paper coupons to recruit other HMSW.

Sex work hot spots constituted the primary sampling units (PSUs) for FSW. Using the different typologies, we established a sampling strategy for each study site. In Mbarara we randomly selected 12 PSUs consisting mainly of streets, lodges and bars/clubs, whereas in Kampala we randomly selected 20 PSUs consisting of streets, lodges, clubs/bars and brothels. Between 6 and 11 participants were enrolled from each PSU at each study site. Sampling began with two FSW at each PSU, who were identified through key informants (managers, club/bar maids and procurers). They were provided information about the study and given three paper coupons to distribute to potential study respondents in their networks. No incentives were given for coupon distribution. For both HMSW and FSW, the coupon contained an identification number, contact information of the research team and duration of the survey in the study site. FSW and HMSW who presented a coupon after verification, and met the eligibility criteria, were consented to participate in the study and completed an interviewer-administered questionnaire. After the interview, each enrollee was given two coupons to recruit more two potential respondents. Face-to-face interviews were conducted by the same interviewer at each site in order to minimize multiple presentation.

Before the interview, respondents were asked about prior or current use of ART. Respondents who were taking ART or knew their HIV status were excluded from the study. All study respondents received information about STI and HIV screening. The study was approved by the Higher Degrees Research and Ethics Committee, School of Public Health, Makerere University and the Uganda National Council for Science and Technology (HS 2403). All respondents provided written informed consent in their language of preference.

### Study variables

The primary outcome variable was recent syphilis (≤ 6 months) and HIV (≤ 12 month) testing. Respondents were asked if they had ever taken a serological syphilis and HIV test (Yes = 1, No = 2) and the number of times they had tested in the past 12 months. A 65-item questionnaire validated among FSW in Benin [31] was used to obtain data on the primary outcome and the major explanatory variables–intention (INT), attitude (ATT), self-efficacy (SE), descriptive norm (MN) and social influences (SI)–which were psychosocial constructs derived from Integrated Change Model [32]. The Integrated Change Model is used to explain motivational and behavioral change. It states that personal overt and covert behaviors are determined by individual motivation or intention to carry out HIV or syphilis testing [32], and that motivation is determined by attitudes, social influences and self-efficacy. We used integrated change model constructs to derive the psychosocial variables, i.e., intentions, attitudes, social influences, norms and self-efficacy of 3-monthly and 6-monthly HIV testing.

#### Intention

Intention in this study was defined as readiness to take a syphilis serological test (SYP_INT) in the next 3 months and an HIV (HIV_INT) test in the next 6 months by asking respondents three items for each infection, e.g., “Are you going to be screened for syphilis in the next 3 months”? Answers on a 6-point Likert scale ranged from 1 =’Strongly disagree’ to 6 = ‘Strongly agree’.

#### Attitude

Attitudes towards 3-monthly syphilis testing (SYP_ATT) were assessed through three items by asking respondents to what extent they agreed with statements such as, “For you getting tested for syphilis every 3 months, would reduce your risk of contracting HIV”. Answer options on a 6-point Likert scale ranged from 1 = ‘Strongly disagree’ to 6 = ‘Strongly agree’. Attitudes towards 6-monthly HIV testing (HIV_ATT) were assessed by asking respondents five items about the benefits and disadvantages of being tested for HIV every 6 months.

#### Descriptive norms

Descriptive norms regarding 3-monthly syphilis testing (SYP_MN) were assessed by asking respondents three questions about whether FSW or HMSW thought it was their moral obligation to test, and how often they thought their peers were testing for syphilis every 3 months. Respondents were asked to what extent they agreed with statements like “Being tested for syphilis is a normal routine that many FSW or HMSW practice?” Answers on a 6-point Likert scale ranged from 1 = ‘Strongly disagree’ to 6 = ‘Strongly agree’. Three items on the same scale were used to assess descriptive norms to 6-monthly HIV testing (HIV_MN) (e.g. “Based on what you know about your fellow FSW or HMSW and the practice of HIV testing, how many of them are being tested every 6 months?” Answering options ranged from 1 = ‘None’ to 6 = ‘All’.

#### Self-efficacy

Self-efficacy for 6-monthly HIV testing (HIV_SE) was assessed by seven questions about their perceived level of confidence and ease of seeking 6-monthly HIV testing. Items included a range of barriers including stigma, discrimination, fear of positive results, privacy and confidentiality. Questions included, “Do you feel able to go for an HIV test every 6 months, even if you are afraid of receiving a positive result?” Answering options on a 6-point Likert scale ranged from 1 = ‘Strongly disagree’ to 6 = ‘Strongly agree’. Self-efficacy for 3-monthly syphilis testing (SYP_SE) was assessed by one statement of whether they feel able to go for a serological syphilis test every 3 months.

#### Social influence

Social norms regarding 6-monthly HIV testing (HIV_SI) were assessed on a 6-point scale (1 = ‘Disapprove strongly’ to 6 = ‘Approve strongly’) by asking respondents two questions on whether referent others (fellow FSW or HMSW and regular partners/clients) approved or expected them to test for HIV every 6 months. We also obtained socio-demographic data on education, marital status and dependents.

#### Statistical analysis

Analyses were performed using Stata version 12.0 (StataCorp, College Station, TX). We used Cronbach’s alpha to evaluate the reliability of items in the questionnaire that were used to assess the major explanatory variables [33]. We computed a scale dimension for each component (SYP_INT, HIV_INT, SYP_ATT, HIV_ATT, SYP_MN, HIV_MN, and HIV_SE) by summing up Likert scores of individual items. The item for social influence (HIV_SI) had a non-reliable scale and was excluded from further analysis. Ordinal data were descriptively summarized using the scale median. Respondents were considered to score high on a component if their total score on the scale was above the scale median, while those with scores below the scale median were considered to score low on a component. Likert scale scores of 1–4 were considered low and scores of 5–6 were high for individual question item. Frequency distributions and proportions were used to describe demographic characteristics, condom use and syphilis and HIV testing behaviors of MSW and FSW. Pearson’s Chi square (χ^2^) tests were used to examine differences between major explanatory variables and sex. Student *t* test was used to ascertain if FSW and HMSW differed with regard to age, duration in sex work, and number of children or dependents. We evaluated predictors of self-reported frequency of recent HIV and syphilis testing (count data) using negative binomial regression (for overdispersed count data). We used a likelihood ratio test of alpha = 0 (dispersion parameter) to assess model fit and found no evidence of over-dispersion. Crude and adjusted prevalence ratios (PR) and 95% confidence intervals (CI) were estimated. We considered two-sided p-values of 0.05 or less statistically significant.

## Results

### Population Characteristics

A total of 340 SW took part in the study, of whom 100 (29.4%) were HMSW. The median age was 27 years [interquartile range (IQR) 25–30] for HMSW and 26 years [IQR], (23–29) for FSW (Table 1). The median duration of sex work was 36 and 30 months for MSW and FSW, respectively. Relative to FSW, most HMSW (89%) had obtained secondary or high education. A higher proportion of FSW (82%) reported having children compared to 64% of HMSW. Most HMSW (70%) had never married and only 11% engaged in full time sex work. They preferred female clientele aged 35 years or greater, whom they solicited through procurers, dating sites, social media and recreational venues. By contrast, FSW solicited clients on the street and in lodges, clubs, bars and brothels. Most HMSW (61%) serviced one to two clients per week compared with 92% of FSW who had five or more clients per week. Condom use at last sex was reported by only 16% of HMSW compared with 66% of FSW (Table 1). Similarly, consistent condom use was reported by only 10% of HMSW compared with 63% of FSW.

**Table 1:** Sex Worker Socio-demographic Characteristics

Variable	Male (N = 100) N (%) or median (IQR)	Female (N = 240) N (%) or median (IQR)	p-value
Age (years)	27 (25–30)	26 (23–29)	0.22
Duration of sex work (months)	36 (24–60)	30 (13–48)	0.52
Biological children	1 (0–2)	1 (1–2)	0.01
Other dependants	1 (0–2)	1 (1–2)	0.76
Education level			0.001
None	0 (0)	25 (10.4)	
Primary	11 (11.0)	108 (45)	
Secondary	54 (54.0)	100 (41.7)	
Higher education	35 (35.0)	7 (2.9)	
Marital status			0.001
Married	7 (7.0)	12 (5)	
Separated	23 (23.0)	83 (34.6)	
Widow	0 (0)	9 (3.8)	
Never married	70 (70.0)	136 (56.4)	
Solicitation of clients			N/A
Street	0 (0)	154 (31.6)	
Home	0 (0)	9 (1.9)	
Lodge	0 (0)	171 (35.2)	
Bar/Club	40 (10.2)	123 (25.3)	
Brothel	0 (0)	29 (6.0)	
Dating site	55 (14.0)	0 (0)	
Procurer	60 (15.3)	0 (0)	
Dating site	48 (12.2)	0 (0)	
Social media (Facebook, Instagram & WhatsApp)	50 (12.8)	0 (0)	
Recreation venues (swimming pool, saunas, beaches & hotels)	49 (12.5)	0 (0)	
Escort service	30 (7.7)	0 (0)	
Client referrals	40 (10.2)	0 (0)	
Workplace (hair salon, car washing bay)	20 (5.1)	0 (0)	
Description of sex worker			0.001
Full time, no other source of income	16 (16.0)	147 (61.2)	
Full time, supplements income	11 (11.0)	24 (10.0)	
Part time, have other sources of income	69 (69.0)	66 (27.5)	
Part time, student	4 (4.0)	3 (1.3)	
Average number of clients per week			0.001
1–2	61 (61.0)	0 (0)	
3–4	29 (29.0)	20 (8.3)	
≥ 5	10 (10.0)	220 (91.7)	
Age of female clients (years)			
20–29	12 (6.1)	N/A	
30–34	42 (21.2)	N/A	
35–39	65 (32.8)	N/A	
≥ 40	79 (39.9)	N/A	
Mobility			0.02
Work only in this town	39 (39.0)	185 (77.1)	
Move regularly to other towns in Uganda	52 (52.0)	50 (20.8)	
Travel outside Uganda	9 (9.0)	5 (2.1)	

### Construct reliability

The degree of internal consistency among question items was assessed using Cronbach’s alpha. The Cronbach’s α coefficients for SYP_INT, HIV_INT, SYP_ATT, HIV_ATT, SYP_MN, HIV_MN, HIV_SE and HIV_SI were 0.96, 0.95, 0.7, 0.85, 0.7, 0.78, 0.89 and 0.39, respectively.

### Syphilis and HIV testing

Compared to FSW (62.5%), self-report of ever testing for syphilis among HMSW was low (32%), as was testing in the prior 6 months (6% vs. 19%) (Table 2). Reasons cited by HMSW for not testing—fear (33%), not feeling sick (30%) and not beneficial (20%)–differed from FSW, for whom not being aware (40%), never thought about it (35%) and have no signs of illness (20%) influenced non-testing behaviors. Similarly, preferences for syphilis testing venues differed by sex, with 78% of HMSW preferring private clinics while FSW tested at public health clinics (49%), private clinics (35%) or during outreach campaigns (10%). Self-reported HIV testing in the prior 12 months was low among HMSW compared to FSW (50% vs 86%), as was ever testing for HIV (80% vs. 96%). In contrast to syphilis testing, HMSW preferences for HIV testing were diverse: public clinics (34%), private clinics (29%), outreach campaigns (23%), and self-testing (8%). FSW preferred to test at public (53%) or private clinics (45%).

**Table 2:** Condom use and STI/HIV testing behaviors

Variable	Male (N = 100) N (%)	Female (N = 240) N (%)	p-value
Condom use at last sexual intercourse			0.001
Yes	34 (34.0)	201 (83.8)	
No	66 (66.0)	39 (16.2)	
Condom use practices			0.001
Always	10 (10.0)	152 (63.0)	
Sometimes	90 (90.0)	88 (37.0)	
Ever had a serological test for Syphilis/STI			0.001
Yes	32 (32.0)	150 (62.5)	
No	68 (68.0)	90 (37.5)	
Syphilis/STI serological testing frequency in the past 12 months			0.001
00	81 (81.0)	114 (47.5)	
1	10 (10.0)	49 (20.4)	
2	3 (3.0)	30 (12.5)	
3	4 (4.0)	38 (15.8)	
≥ 4	2 (2.0)	9 (3.8)	
Ever tested for HIV			
Yes	80 (80.6)	230 (96.0)	0.02
No	20 (20.0)	10 (4.0)	
HIV serological testing frequency in the past 12 months			0.001
00	48 (50.0)	34 (14.0)	
1	8 (8.3)	46 (19.2)	
2	9 (9.4)	52 (21.7)	
3	20 (20.8)	75 (31.3)	
≥ 4	11 (11.5)	33 (13.8)	
History of HIV			N/A
I don’t know	38 (38.0)	8 (3.3)	
No	59 (59.0)	232 (96.7)	
Yes	3 (3.0)		
History of Syphilis			N/A
I don’t know	54 (54.0)	12 (5.0)	
No	20 (20.0)	91 (37.9)	
Yes	26 (26.0)	137 (57.1)	
History of Gonorrhoea			
I don’t know	4 (4.0)	13 (5.4)	
No	28 (28.0)	175 (72.9)	
Yes	68 (68.0)	52 (21.7)	

### Psychosocial influences of regular syphilis and HIV testing

We found that compared to FSW, HMSW had low intentions or attitudes towards 3-monthly Syphilis or 6-montthly HIV testing (Table 3). Relative to FSW, a low proportion of HMSW had intentions to test for syphilis in the next 3 months (19% vs. 63%), test for HIV in the next 6 months (24% vs. 66%), believe in the benefits of regular testing for syphilis (29% vs. 65%) or HIV (11% vs. 75%), have self-efficacy to seek regular syphilis (40% vs. 84%) or HIV testing (38% vs. 60%) or perceive that regular syphilis (33% vs. 68%) or HIV testing (19% vs. 70%) was normative for SW (Table 4).

**Table 3:** Description of HMSW and FSW with regard to attitudes, social influences, descriptive norms and self-efficacy towards 3-mpnthly syphilis and 6-monthly HIV testing

Variable	Male (N = 97)	Female (N = 240)	p-value
SYP_INT			
You intend to go for a serological syphilis test during the next 3 months			0.001
Agree + Strongly agree	5 (5.2)	106 (44.2)	
Disagree + Strongly disagree	92 (94.8)	134 (55.8)	
You are going to be tested for syphilis in the next 3 months			0.001
Agree + Strongly agree	6 (6.2)	108 (45.0)	
Disagree + Strongly disagree	91 (93.8)	132 (55.0)	
How would you rate your chances that you will take a serological syphilis test in the next 3 months?			0.001
High + Very high	5 (5.2)	86 (35.8)	
Low + Very low	92 (94.8)	154 (64.2)	
HIV_INT			
You intend to go for HIV testing during the next 3 months			0.001
Agree + Strongly agree	34 (35.0)	195 (81.5)	
Disagree + Strongly disagree	63 (65.0)	45 (18.8)	
You are going to be tested for HIV in the next 3 months			
Agree + Strongly agree	28 (28.9)	186 (77.5)	
Disagree + Strongly disagree	69 (71.1)	54 (22.5)	
How would you rate your chances of being tested for HIV in the next 3 months?			0.001
High + Very high	29 (29.9)	162 (67.5)	
Low + Very Low	68 (70.1)	78 (32.5)	
SYP_ATT			
For you to be tested for syphilis every 3 months would be			0.001
Beneficial + Very beneficial	5 (5.2)	132 (55.0)	
Not beneficial + Not very beneficial	92 (94.8)	108 (45.0)	
For you being tested for syphilis and other ulcerative STI every 3 months would reduce your risk of contracting HIV			0.001
Agree + Strongly agree	15 (15.5)	129 (53.7)	
Disagree + Strongly disagree	82 (84.5)	111 (46.3)	
You would access syphilis testing every 3 months			0.001
Agree + Strongly agree	12 (12.4)	97 (40.1)	
Disagree + Strongly disagree	85 (87.6)	143 (59.6)	
HIV_ATT			
For you be tested for HIV every 6 months would be			0.001
Beneficial + Very beneficial	49 (50.5)	203 (84.6)	
Not beneficial + Not very beneficial	48 (49.5)	37 (15.4)	
For you getting tested for HIV every 6 months would allow you to be better informed about your health			0.001
Agree + Strongly agree	29 (29.9)	209 (87.1)	
Disagree + Strongly disagree	68 (70.1)	31 (12.9)	
For you getting tested for HIV every 6 months would help you better protect yourself			0.001
Agree + Strongly agree	20 (20.6)	199 (82.9)	
Disagree + Strongly disagree	77 (79.4)	41 (17.1)	
You would fill proud if you are tested for HIV every 6 months			0.001
Agree + Strongly agree	38 (39.0)	199 (82.9)	
Disagree + Strongly disagree	59 (61.0)	41 (17.1)	
You would access treatment if you tested for HIV every 6 months			
Agree + Strongly agree	41 (42.0)	183 (76.2)	0.001
Disagree + Strongly disagree	56 (58.0)	57 (23.8)	
HIV_SI			
Influence of reference others (approval of HIV testing every 3–6 months)			
Fellow sex workers			0.45
Approve + Strongly approve	22 (22.7)	55 (23.0)	
Disapprove + Strongly disapprove	75 (77.3)	185 (77.0)	
Regular clients			0.001
Approve + Strongly approve	9 (9.3)	105 (43.7)	
Disapprove +Strongly disapprove	88 (90.7)	135 (56.3)	
SYP_MN			
When you are a sex worker it is necessary to go for syphilis testing every 3 months			0.001
Agree + Strongly agree	5 (5.2)	127 (52.9)	
Disagree + Strongly disagree	92 (94.8)	113 (47.1)	
Being tested for syphilis every 3 months is a normal routine than many FSW and HMSW practice			
Agree + Strongly agree	6 (6.2)	57 (23.7)	0.03
Disagree + Strongly disagree	91 (93.8)	183 (76.3)	
Based on what you know about your fellow FSW and HMSW and practice of syphilis testing, how many of them are being tested every 3 months			0.045
Half (50%) + Majority (75%) + all	8 (8.2)	38 (15.8)	
None + Minority (25%)	89 (91.8)	202 (84.2)	
HIV_MN			
When you are a sex worker it is necessary to go for HIV testing every 6			0.001
Agree + Strongly agree	12 (12.4)	203 (84.2)	
Disagree + Strongly disagree	85 (87.6)	38 (15.8)	
Being tested for HIV every 6 months is a normal routine than many FSW and HMSW practice			0.02
Agree + Strongly agree	12 (12.4)	116 (48.3)	
Disagree + Strongly disagree	85 (87.6)	124 (51.7)	
Based on what you know about your fellow FSW/HMSW and practice of HIV testing, how many of them are being tested every 6 months			0.04
Half (50%) + Majority (75%) + all	32 (33.0)	129 (53.8)	
None + Minority (25%)	65 (67.0)	111 (46.2)	
SYP_SE			
You are confident you can go and test for syphilis every 3 months			0.01
Agree + Strongly agree	7 (7.2)	102 (42.5)	
Disagree + Strongly disagree	90 (92.8)	138 (57.5)	
HIV_SE			
For you to be tested for HIV every 6 months would be			0.02
Easy + Very easy	34 (35.0)	125 (52.0)	
Difficult + Very difficult	63 (65.0)	115 (48.0)	
I am confident i can go for HIV testing every 6 months			
Agree + Strongly agree	38 (39.0)	189 (78.8)	0.001
Disagree + Strongly disagree	59 (61.0)	51 (21.2)	
Do you feel able to go for an HIV test every 6 months even if you are afraid of receiving a positive result			0.03
Agree + Strongly agree	34 (35.0)	122 (51.0)	
Disagree + Strongly disagree	63 (65.0)	118 (49.0)	
Do you feel able to go for an HIV test every 6 months despite fear of discrimination and stigma in case of a positive result			0.06
Agree + Strongly agree	38 (39.0)	99 (41.0)	
Disagree + Strongly disagree	59 (61.0)	141 (59.0)	
Do you feel able to go for an HIV test every 6 months, even if you do not know completely if this information will remain confidential			0.03
Agree + Strongly agree	34 (35.0)	101 (42.0)	
Disagree + Strongly disagree	63 (65.0)	139 (58.0)	
If HIV testing is free you will go for HIV testing every 6 months			0.001
Agree + Strongly agree	36 (37.1)	191 (79.6)	
Disagree + Strongly disagree	61 (62.9)	49 (20.4)	
You would go for HIV testing every 6 months if you know where the services is offered			0.001
Agree + Strongly agree	32 (33.0)	205 (85.4)	
Disagree + Strongly disagree	65 (67.0)	35 (14.6)

**Table 4:** Median scores of scale dimensions for HMSW and FSW

Variable	Male (N = 97)	Female (N = 240)	p-value
SYP_INT			0.001
Score ≥ 9, median	18 (19)	152 (63.3)	
Score < 9, median	79 (81)	88 (36.7)	
HIV_INT			0.001
Score ≥ 15, median	23 (23.7)	158 (65.8)	
Score < 15, median	74 (76.3)	82 (34.2)	
SYP_ATT			0.001
Score ≥ 8, median	28 (28.9)	155 (64.6)	
Score < 8, median	69 (71.1)	85 (35.4)	
HIV_ATT			0.001
Score ≥ 25, median	11 (11.3)	180 (75.0)	
Score < 25, median	86 (88.7)	49 (25.0)	
SYP_MN			0.001
Score ≥ 8, median	32 (33.0)	162 (67.5)	
Score < 8, median	65 (67.0)	78 (32.5)	
HIV_MN			0.001
Score ≥ 16, median	18 (18.6)	167 (69.6)	
Score < 16, median	79 (81.4)	73 (30.4)	
HIV_SE			0.001
Score ≥ 30, median	37 (38.1)	143 (59.6)	
Score < 30, median	60 (61.9)	97 (40.4)	
SYP_SE			0.001
Score ≥ 3, median	39 (40.0)	202 (84.2)	
Score < 3, median	58 (60.0)	38 (15.8)	

*SYP_INT* intention to seek 3-monthly syphilis serological testin, *HIV_INT* intention to seek 6-monthly HIV testing, *SYP_ATT* attitude towards 3-monthly syphilis testing, *HIV_ATT* attitude towards 6-monthly HIV testing, *SYP_MN* perceived prevalence of the practice of 3-monthly testing, *HIV_MN* perceived prevalence of the practice of 6-monthly HIV testing, *HIV_SE* self-efficacy to seek 6-monthly HIV testing

### Associations with syphilis and HIV testing

Next, we examined factors associated with testing for syphilis and HIV in the prior 6 and 12 months, respectively. In multivariate analysis after adjustment for age, level of education, marital status, study site, attitudes to testing and condom use practices, attainment of higher education [adjusted prevalence ratio (aPR) 1.66; 95% confidence interval (CI) 1.09–2.50; p = 0.02], poor intention to seek HIV testing (aPR 1.64; 95% CI 1.35–2.04; p < 0.001), perception that 6-monthly HIV testing was not common (aPR 1.33; 95% CI 1.09–1.67; p = 0.007) and poor self-efficacy (aPR 1.41; 95% CI 1.12–1.79; p = 0.005) were associated with HIV non-testing (Table 5).

**Table 5:** Negative binomial multivariable model for HIV testing in the prior 12 months

	Unadjusted prevalence ratio (PR)			Adjusted prevalence ratio (aPR)		
	PR (SE)	95% CI	p-value	aPR (SE)	95% CI	p-value
Age (years)						
17–19	Reference					
20–24	1.18 (0.3)	0.75–1.84	0.47	1.21 (0.3)	0.78–1.89	0.39
25–29	1.24 (0.3)	0.80–1.92	0.34	1.19 (0.3)	0.76–1.86	0.45
30–34	1.28 (0.3)	0.81–2.03	0.29	1.12 (0.3)	0.69–1.82	0.65
35 +	1.07 (0.2)	0.63–1.79	0.80	0.93 (0.2)	0.54–1.60	0.79
Level of education						
None	Reference					
Primary	0.97 (0.2)	0.71–1.32	0.83	1.04 (0.2)	0.76–1.43	0.79
Secondary	0.89 (0.1)	0.65–1.20	0.44	1.04 (0.2)	0.755–1.44	0.81
Higher education	1.08 (0.2)	0.76–1.53	0.65	1.66 (0.3)	1.09–2.50	*0.02*
Marital status						
Married	Reference					
Separated	1.24 (0.2)	0.84–1.83	0.28	1.43 (0.3)	0.95–2.14	0.08
Widow	1.24 (0.4)	0.69–2.23	0.42	1.27 (0.4)	0.69–2.35	0.45
Single	1.00 (0.2)	0.68–1.49	0.96	1.22 (0.3)	0.82–1.83	0.33
Have a regular boyfriend	1.36 (0.3)	0.92–2.00	0.13	1.35 (0.3)	0.90–2.03	0.14
Location						
Kampala	Reference					
Mbarara	0.95 (0.1)	0.81–1.11	0.53	1.02 (0.1)	0.86–1.22	0.81
Sex						
Male						
Female	1.56 (0.1)	1.29–1.89	*0.001*	0.97 (0.2)	0.70–1.33	0.84
Female	1.56 (0.1)	1.29–1.89	*0.001*	0.97 (0.2)	0.70–1.33	0.84
Condom use						
Yes	Reference					
No	0.66 (0.1)	0.54–0.79	*0.001*	0.86 (0.1)	0.69–1.08	0.19
HIV_INT						
Score ≥ 15, median	Reference					
Score < 15, median	0.46 (0.04)	0.39–0.55	*0.001*	0.61 (0.1)	0.49–0.74	*0.001*
HIV_ATT						
Score ≥ 25, median	Reference					
Score < 25, median	0.55 (0.05)	0.46–0.65	*0.001*	0.88 (0.1)	0.69–1.12	0.30
HIV_MN						
Score ≥ 16, median	Reference					
Score < 16, median	0.54 (0.04)	0.45–0.64	*0.001*	0.75 (0.1)	0.60–0.92	*0.007*
HIV_SE						
Score ≥ 30, median	Reference					
Score < 30, median	0.44 (0.03)	0.37–0.52	*0.001*	0.71 (0.1)	0.56–0.89	*0.005*

*PR*, unadjusted prevalence ration, *aPR* adjusted prevalence ration, *SE* standard error

Likelihood-ratio test of alpha = 0: chibar2 (01) = 0.0e + 00 Prob ≥ chibar2 = 0.500, log likelihood = − 517.0

In the multivariable model adjusting for age, level of education, marital status, and study site, low intention to seek syphilis testing (aPR 3.13; 95% CI 2.13–4.55; p < 0.001), negative testing attitudes (aPR 2.33; 95% CI 1.64–3.33; p = 0.004), perception that regular testing was not normative (aPR 1.59; 95% CI 1.14–2.22; p < 0.001), and low self-efficacy (aPR 2.56; 95% CI 1.35–4.76; p = 0.007) were associated with syphilis non-testing (Table 6).

**Table 6:** Negative binomial multivariable model for syphilis testing in the prior 12 months

	Unadjusted ratio (PR)			Adjusted ratio (aPR)		
	PR (SE)	95% CI	p-value	aPR (SE)	95% CI	p-value
Age (years)						
17–19	Reference					
20–24	1.59 (0.8)	0.62–4.11	0.34	1.54 (0.6)	0.73–3.27	0.26
25–29	1.59 (0.8)	0.62–4.05	0.33	1.38 (0.5)	0.65–2.91	0.40
30–34	1.49 (0.7)	0.56–3.97	0.42	1.12 (0.4)	0.49–2.52	0.78
35 +	1.26 (0.7)	0.42–3.78	0.68	1.00 (0.5)	0.41–2.46	0.98
Level of education						
None	Reference					
Primary	0.88 (0.3)	0.47–1.65	0.69	0.98 (0.2)	0.62–1.56	0.95
Secondary	0.75 (0.2)	0.40–1.39	0.35	0.97 (0.2)	0.61–1.55	0.91
Higher education	0.71 (0.3)	0.33–1.49	0.35	1.39 (0.4)	0.73–2.66	0.32
Marital status						
Married	Reference					
Separated	0.84 (0.3)	0.40–1.75	0.63	1.23 (0.3)	0.71–2.13	0.45
Widow	1.17 (0.7)	0.37–3.73	0.79	1.63 (0.7)	0.71–3.74	0.25
Single	0.82 (0.3)	0.39–1.69	0.59	1.23 (0.3)	0.72–2.11	0.45
Have regular boy/girlfriend	1.12 (0.4)	0.53–2.34	0.77	1.34 (0.4)	0.79–2.27	0.28
Study site						
Kampala	Reference					
Mbarara	0.69 (0.1)	0.49–0.97	0.03	1.06 (0.2)	0.79–1.41	0.71
Sex						
Male	Reference					
Female	3.0 (0.6)	1.99–4.55	0.001	1.08 (0.3)	0.64–1.84	0.75
Condom use						
Yes	Reference					
No	0.53 (0.1)	0.37–0.78	0.001	0.89 (0.2)	0. 64–1.27	0.54
SYP_INT						
Score ≥ 9, median	Reference					
Score < 9, median	0.16 (0.02)	0.11–0.22	0.001	0.32 (0.1)	0.22–0.47	*0.001*
SYP_ATT						
Score ≥ 8, median	Reference					
Score < 8, median	0.22 (0.03)	0.15–0.31	0.001	0.43 (0.1)	0.30–0.61	*0.001*
SYP_MN						
Score ≥ 8, median	Reference					
Score < 8, median	0.28 (0.04)	0.19–0.39	0.001	0.63 (0.1)	0.45–0.88	*0.007*
SYP_SE						
Score ≥ 3, median	Reference					
Score < 3, median	0.12 (0.03)	0.064–0.21	0.001	0.39 (0.1)	0.21–0.74	*0.004*

Likelihood-ratio test of alpha = 0: chibar2(01) = 0.17 Prob ≥ chibar2 = 0.340, Log likelihood = − 344.47

## Discussion

To our knowledge, this is the first study to evaluate HIV and syphilis testing among HMSW in Uganda. In this cross-sectional study, the majority of HMSW had attained higher education but had lower testing rates for syphilis and HIV and less condom use than FSW. HMSW preferred testing in private health facilities whereas FSW preferred public health facilities. Among HMSW, HIV non-testing was associated with higher education, poor self-efficacy, and poor testing norms and perceptions. Syphilis non-testing was associated with negative testing attitudes, low self-efficacy and low intention to seek testing.

We found that 50% of HMSW reported not testing for HIV in the past 12 months. Non-testing for HIV was common in prior studies from Asia and Africa [34–36]. A study of HMSW in Singapore found that only 27% had tested for HIV or STIs in the past 6 months [34]. In China, only 48.6% of MSW who have sex with men had tested for HIV [35, 36]. In Kenya, a study found low prevalence (26%) of recent HIV testing among MSW [37]. Work done in the Netherlands also found that majority of HMSW (63%) reported no recent history of HIV or STI testing compared with 32% of FSW [38]. However, most of these studies focused on MSW whose clients were male [35–37]. The higher HIV testing rate (86%) we found could be due to targeted HIV testing programs for FSW in Uganda [39–41]. Similar findings were reported from a recent study in Uganda where 86% of FSW reported taking an HIV test in the prior 12 months [29]. HMSW are hidden key sub-population largely invisible to HIV programs [38]. Entrenched social stigma, healthcare discrimination and criminalization of sex work limit access to, and uptake of, testing services [42, 43]. Additionally, HMSW may not identify as sex workers nor perceive themselves to be at risk of HIV and other STIs [20, 38, 44]. These factors may account for the low testing norms, low self-efficacy and poor uptake of HIV testing we observed. Although higher levels of education have been associated with better health seeking behaviours [45, 46], better educated HMSW in our study were less likely to test for HIV than FSW perhaps because of lack of HIV services targeted to HMSW.

We found low syphilis testing rates among HMSW and FSW which is consistent with prior studies of sex workers in Uganda, and elsewhere, that reported low syphilis testing behaviors [3, 20, 30, 47, 48]. Syphilis is a less stigmatized disease than HIV in Uganda [49]. One study found that the perception of syphilis as a genetic (inherited) disease was a barrier to testing among men [50]. Our finding that testing rates were lower for syphilis than HIV could be explained by social and individual perceptions of these diseases. Unlike HIV, syphilis is not perceived as a significant threat to personal health in this setting [51]. Additionally, FSW are more likely to receive information about syphilis testing during moonlight HIV counselling and testing (HCT) outreach campaigns and antenatal care; dual syphilis and HIV testing is standard of care in antenatal clinics in Uganda [39, 52]. Testing attitudes and intentions are influenced by knowledge and personal evaluation of the merits and demerits of regular syphilis testing [53, 54]. Lack of knowledge, stigma and poor attitudes of health workers are barriers to utilization of STI services [55]. The low intentions, negative attitudes and poor testing norms we observed among HMSW could suggest lack of comprehensive knowledge of syphilis, the benefits of regular testing, and barriers to testing including stigma, discrimination and perceived attitudes of providers [44]. These findings are consistent with studies showing that psychosocial factors including intentions, attitudes, norms and self-efficacy influence STI and HIV testing behaviors [56]. They may also result from policy and programmatic focus on HIV and frequent stock outs of syphilis test kits in Uganda. Compared to HIV, where national testing guidelines [57] target the general population, syphilis guidelines focus on pregnant women [39]. These policy choices could limit HMSW access to regular syphilis testing unlike FSW who are targeted during testing campaigns or antenatal care [39, 41]. Scaling up point-of-care (POC) testing for HIV and syphilis increases uptake of testing services by sex workers [58] and enables early treatment of both diseases [59, 60].

The strengths of our study include being the first study (to our knowledge) to evaluate HIV and syphilis testing among HMSW in Uganda and use of the integrated change model to guide the design and analysis of major explanatory variables with reliable item statements (Cronbach’s α ≥ 0.7). Our study has limitations. The study design was cross-sectional, and our findings do not account for time trends in testing behaviors. Participants recruited from two large urban centers may not be representative of all HMSW in Uganda. Social desirability and recall bias may have influenced self-report of HIV and syphilis testing behaviors. Nevertheless, studies with larger sample sizes and longer duration of follow up have reported similar findings.

In conclusion, non-testing for HIV and syphilis was common among HMSW in Uganda. These data inform HIV and STI programming for sex workers which should scale-up dual HIV and syphilis POC testing for HMSW. Future studies should evaluate strategies to increase testing uptake in this neglected sub-population of sex workers.

## Data Availability

The datasets used during the current study are available from the corresponding author on request. The questionnaire is included as supplementary information.

## Abbreviations

FSW: Female sex workers
HIV: Human immunodeficiency virus
IQR: Interquartile range
MSW: Male sex workers
HMSW: Heterosexual male sex workers
PSU: Primary sampling unit
STI: Sexually transmitted infection
WHO: World Health Organization
